# Neuromorphic Signal Filter for Robot Sensoring

**DOI:** 10.3389/fnbot.2022.905313

**Published:** 2022-06-13

**Authors:** Luis M. García-Sebastián, Victor H. Ponce-Ponce, Humberto Sossa, Elsa Rubio-Espino, José A. Martínez-Navarro

**Affiliations:** Instituto Politécnico Nacional, Centro de Investigación en Computación, Mexico City, México

**Keywords:** neuromorphic, filter, CMOS, low-frequency, sensoring

## Abstract

Noise management associated with input signals in sensor devices arises as one of the main problems limiting robot control performance. This article introduces a novel neuromorphic filter model based on a leaky integrate and fire (LIF) neural model cell, which encodes the primary information from a noisy input signal and delivers an output signal with a significant noise reduction in practically real-time with energy-efficient consumption. A new approach for neural decoding based on the neuron-cell spiking frequency is introduced to recover the primary signal information. The simulations conducted on the neuromorphic filter demonstrate an outstanding performance of white noise rejecting while preserving the original noiseless signal with a low information loss. The proposed filter model is compatible with the CMOS technology design methodologies for implementing low consumption smart sensors with applications in various fields such as robotics and the automotive industry demanded by Industry 4.0.

## 1. Introduction

The term neuromorphic, coined by Mead (1990), refers to Very Large Scale of Integration (VLSI) systems aiming to reproduce biological neuron behaviors. Neuromorphic computing platforms are relatively simple regarding the number of active elements (transistors) compared to complex traditional digital units (microprocessors) to replicate brain-like responses. Today, the convergence of electronics, computing science, and neuroscience offers bountiful inspiration to explore novel hardware structures, algorithms, and innovative ways to process information more efficiently, maintaining low levels of energy waste and material use (Schuman et al., 2017). One of the most remarkable contributions of this inter-discipline convergence is the conception of spiking neurons (SN), also called the third generation of artificial neurons (Maass, 1997). The main difference concerning previous generations is the inclusion of temporal information in the computing process, and this feature offers the possibility to process signals efficiently with variations across time. Unfortunately, the large-scale modeling of SN units is limited due to the high computational cost involved in solving numerically the whole set of differential equations representing each SN unit. Therefore, the design and implementation of these units are more convenient at the silicon plane and in the analog domain to overpass this vast amount of numerical computation effort.

Traditional analog filters are designed based on scaling specific frequency domain signal components and attenuating the rest. This approach has been proven effective with noise that is primarily out of signal frequency range. However, linear filters cannot clear noisy signals when disturbance affectation is in the same frequency range as primary signal. In this context, digital filters, especially average filter techniques, take precedence at the cost of resources expense. For this reason, some filter proposals based on the use of several SNs have been made (Orchard et al., 2021; Sharifshazileh et al., 2021). Generally, integrated circuits hosting neuromorphic implementations possess an inherent capacity to extract primary features of given entries since integrals tied to the SN model can be interpreted as the average value operator on a time window. Thus, neuromorphic systems allow average filtering while retaining the benefits of analog circuits.

## 2. Methods

### 2.1. Neural Circuit

**Figure 1:**
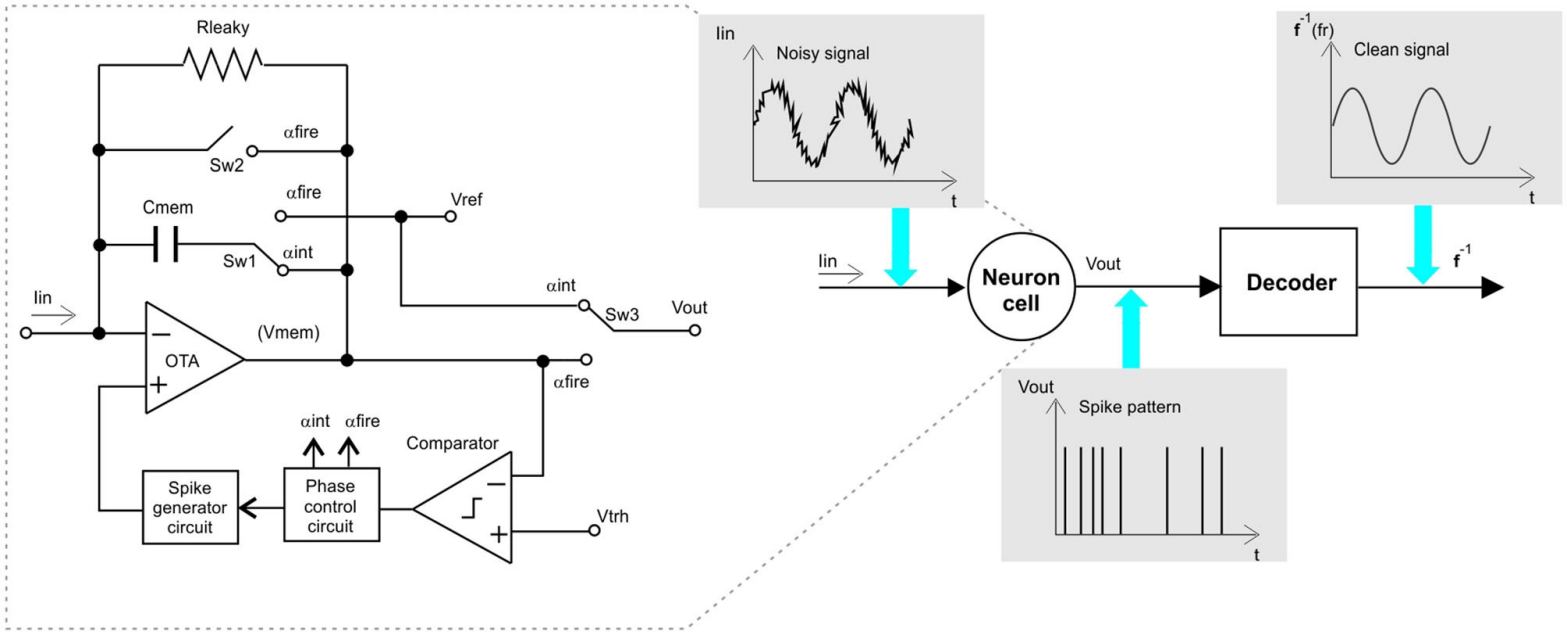
Neuromorphic filter architecture, showing a spiking neuron CMOS circuit implementation, see Wu et al. (2015).

There are several proposals reported on analog implementations of neural model circuits (Abbott, 1999; Wijekoon and Dudek, 2008; Zamarreño-Ramos et al., 2011; Wu et al., 2015; Zare et al., 2021). Throughout the development of this study, the leaky integrate and fire neuron (IFN) circuit, proposed in Wu et al. (2015) was used, as seen in Figure 1. However, the proposed methodology could be easily adapted to work with other neural circuit models. This proposal is divided into two main parts, a leaky, current integrator circuit (LI) to emulate the behavior of a neuron during the period of depolarization and a reset engine that returns the output voltage of the operational amplifier (*V*_*mem*_) to a reference voltage level (*V*_*Ref*_). It also assumes the generation of a convenient spike shape, compliant with memristors technology to allow weight adjustment during the learning phase.

Wu's neural circuit shown in Figure 1 operates in the integration and firing mode. At the integration mode, the OPAMP works as a leaky integrator, over the current, *I*_*in*_, flowing at its negative input. At the integration mode, the voltage level at the output of the OPAMP decreases until it reaches a determined threshold voltage, *V*_*thr*_. The comparator circuit compares the membrane voltage, *V*_*mem*_, with *v*_*thr*_, to generate a signal activating the Phase Control block when the descending *V*_*ref*_ reaches *V*_*thr*_. At this moment, the Phase Control block commands the Spike Generator block to initiate a spike event with a predefined waveform and it changes the control signals, α_*fire*_ to ON state, while α_*int*_ to OFF state. These control signals are complementary. The neural circuit is reconfigured by the current states of α_*fire*_ and α_*int*_. If α_*int*_ is ON, the OPAMP works in the integration mode, if α_*fire*_ is ON the neuron is in fire mode. During integration mode the neuron output *V*_*out*_ is set in *V*_*ref*_, at the same time, the spike generator block must hold a *V*_*ref*_ at the positive OPAMP input, which is buffered at the negative OPAMP input. In the firing mode, *V*_*out*_, is connected to *V*_*mem*_, generating a spike event, feedbacking, *V*_*mem*_ to the negative OPAMP input. At the end of the firing mode, *C*_*mem*_ is reset to a *V*_*ref*_, potential.

The equivalent model of the LI section is presented in Equation (1).


(1) $$\begin{array}{r} \frac{dV_{mem}}{dt}=\frac{V_{p}-V_{mem}\left(t\right)}{R_{leak}C_{mem}}-\frac{I_{in}}{C_{mem}} \end{array}$$

Where *V*_*p*_ is the voltage objective, while the circuit is in integration mode *V*_*p*_ corresponds to *V*_*thr*_.

Wu's circuit functioning could still be simplified to implement the proposed methodology, performing a noise signal filtering process. The simplification consists of establishing constant delays before switching states and restarting the integration phase. It imposes a period of neuron inactivity corresponding to the refractory period, seen in biological brains. This behavior is modeled as shown below:


$$\begin{array}{c} \begin{array}{l} \;if\;V_{mem}\approx V_{thr}\\ \begin{array}{c} then\;V_{p}\leftarrow V_{ref},\;R_{leak}\leftarrow 1,\;I_{in}\leftarrow 0 \end{array} \end{array} \end{array}$$

Once the neuron is in the refractory period, it maintains its state for a predefined period, after which it returns to the previous (integration) state.

### 2.2. Tuning Curves

**Figure 2:**
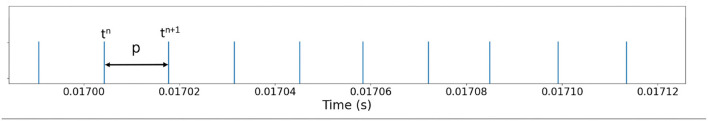
Neuron circuit spike response. Since spike waveform is not needed for the filtering process, spike events are registered as a list of times when the neuron circuit reach threshold voltage. Time elapsed between *t*^*n*^ and *t*^*n*+1^ is denoted as *p*.

Since spike trains convey information through their timing and any spike-wave produced by neural circuits models are supposed to be identical (Gerstner et al., 2016), the membrane's potential in neuromorphic circuits can be characterized simply by a list of events: *t*^0^, *t*^1^, ..., *t*^*n*^, where 0 ≤ *t*^*i*^ ≤ *T*, with *i* = 0, 1, 2, ..., *n* is the *i*-*th* spike time in an observed period *T* (Dayan and Abbot, 2001). Figure 2 shows a representation of this list.

A simple way to characterize the response of a neuromorphic circuit is by counting the number of peak voltages fired during the presentation of a stimulus (input current). By repeating this operation for a certain number of different stimuli, it is possible to estimate a function, *f*, that describes the relationship between an input current, *I*_*in*_, and a frequency of spikes *fr* (Dayan and Abbot, 2001; Elliasmith, 2013). In this study, an alternative way to estimate the neuron frequency is proposed. Since neuromorphic circuits have no stochastic behavior, it is possible to prove that the same neuron frequency response will always be obtained for a given *I*_*in*_. Therefore, by measuring the time elapsed between the event of two spikes, *p* = *t*^*n*^−*t*^*n*−1^ , the frequency is obtained by using $\frac{1}{p}$.

### 2.3. Mean Value Theorem for Integrals

The time between spikes in the circuit presented in Figure 1 corresponds to the mean value of the input current.

Rearranging elements from Equation (1), we find the next expression.


(2) $$\begin{array}{r} I_{in}=\frac{V_{p}-V_{mem}\left(t\right)}{R_{leak}}-C_{mem}\frac{dV_{mem}}{dt}, \end{array}$$

Equation (2) corresponds to Current Kirchhoff's Law, producing a summation of all the currents at input node, *I*_*in*_ = *I*_*R*_*Leak*__+*I*_*C*_*mem*__, where *I*_*R*_*Leak*__(*t*) is the current across *R*_*Leak*_, which behavior is unknown in advance, thus:


(3) $$\begin{array}{r} I_{in}=I_{R_{Leak}}\left(t\right)-C_{mem}\frac{dV_{mem}}{dt}. \end{array}$$

Now, integrating both sides of Equation (3) with the defined time intervals limits between neural events (spike occurrences) results in Equation (4). Internal values of the neuromorphic units are reset at the end of each neural event,


(4) $$\int_{t^{n-1}}^{t^{n}}I_{in}dt=\int_{t^{n-1}}^{t^{n}}I_{R_{Leak}}(t)dt-\int_{V_{ref}}^{V_{thr}}C_{mem}dV_{mem}$$

Solving integrals on both sides:


(5) $$I_{in}(t^{n}-t^{n-1})=\int_{t^{n-1}}^{t^{n}}I_{R_{Leak}}(t)dt-C_{mem}V_{mem}$$

Where the value *V*_*mem*_ = *V*_*thr*_−*V*_*ref*_ results at the end of the integration period. Equation (5) corresponds to the Mean Value Theorem for integrals (Stewart, 2018). Thus, a constant value of *I*_*in*_ exists such that applied for the time interval, *t*^*n*^−*t*^*n*−1^, equals the value of the current *I*_*R*_*Leak*__(*t*) on the same period. Particularly, *I*_*in*_ can be seen as the mean value of current on the period *t*^*n*^−*t*^*n*−1^ plus a constant value (*C*_*mem*_*V*_*mem*_).

## 3. Proposed Methodology for a Neural Filter Design

**Figure 3:**
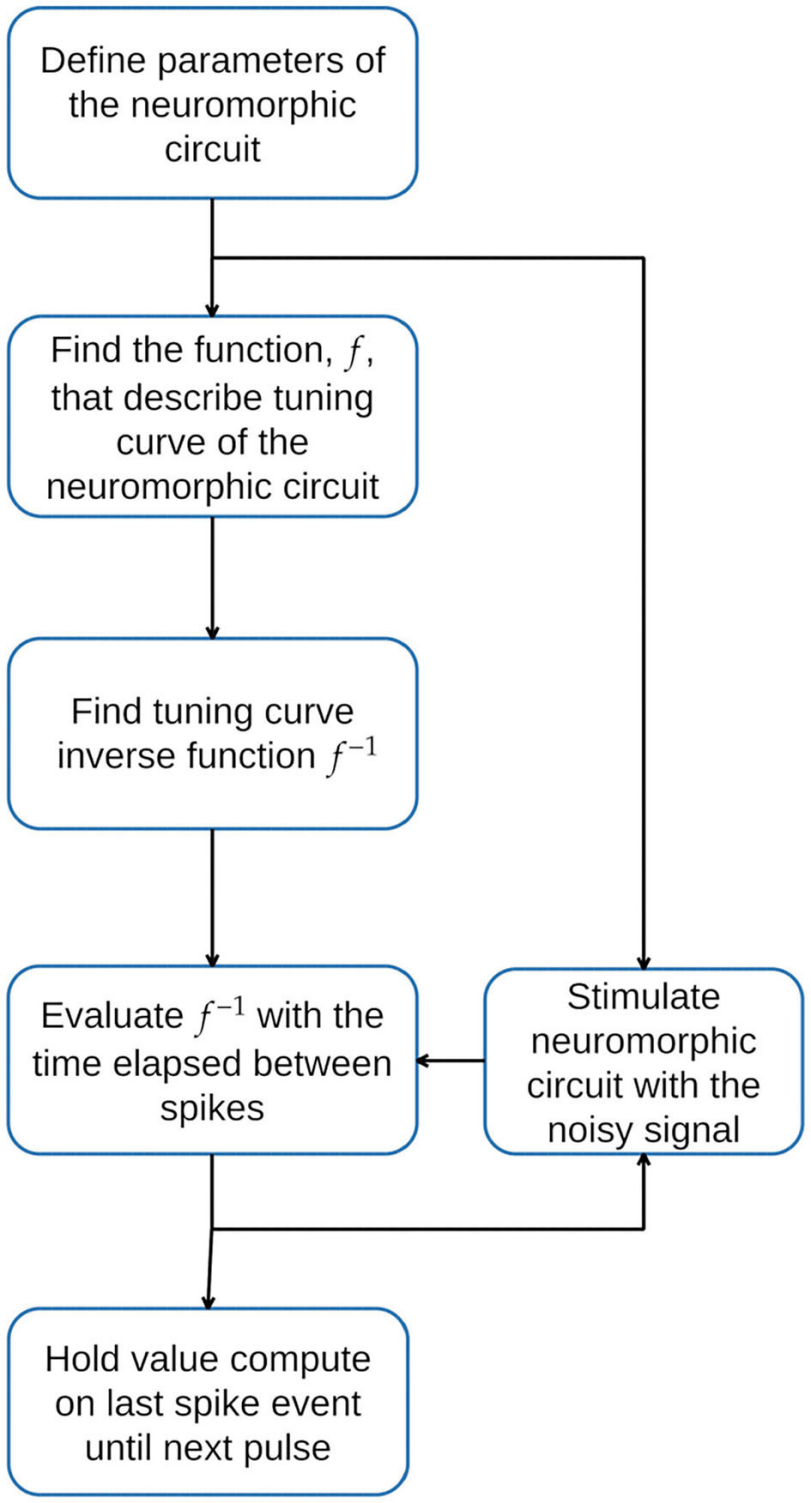
Proposed Methodology. First, define the parameters of the neural circuit. Second, characterize the response to obtain the circuit tuning curve function, *f*, and its inverse function. Finally, stimulate the neural circuit with the noisy input signal and use the time elapsed since the last spike to evaluate the inverse function *f*^−1^. The value computed using *f*^−1^ is held until the next event occurs.

Our proposal consists of using the tuning curve function of the neuromorphic circuit to estimate the *I*_*in*_ value on Equation (5). The methodology proposed to use a neural circuit as a signal filter is depicted in Figure 3.

**Figure 4:**
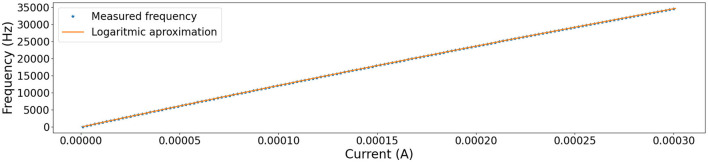
Tuning curve of the used circuit. Marks shows measurements frequencies obtained by sweeping the current *I*_*in*_ of Equation (1) in interval 0−300μ*A*, with electrical and timing parameters: *C*_*mem*_ = 1μ*F*, *R*_*leak*_ = 10*kΩ*, and a refractory period of 10μ*s*. The orange line shows approximation by 7. * refers to the measured frequency of spikes. - refers to measurments approximation made by Equation (6).

The tuning curve for the circuit introduced in Figure 4, is obtained by sweeping the current *I*_*in*_ of Equation (1) between a current interval ∈[0, 300]μ*A*, considering the following electrical and timing parameters: *C*_*mem*_ = 1μ*F*, *R*_*leak*_ = 10*kΩ*, and a refractory period of 10μ*s*, we proceed to measure the time elapsed between potential membrane spikes. The below equation is proposed as a prototype to estimate function *f*.


(6) $$\begin{array}{r} f\left(I_{in}\right)=ln\left(I_{in}+a\right)b-c \end{array}$$

Parameters *a* = 1724.8761, *b* = 21.6051, *c* = −161.1285, are determined using nonlinear least squares curve fitting (Virtanen et al., 2020).

Because Equation (6) is invertible, we can take two produced spikes and calculate the current in the elapsed period between spikes. That is to say, *f*^−1^ computes the equivalent input current value in the system (*I*_*in*_).


(7) $$\begin{array}{r} f^{-1}\left(fr\right)=I_{in}\left(fr\right)=\beta \left[e^{\frac{fr-c}{b}}-a\right] \end{array}$$

**Figure 5:**
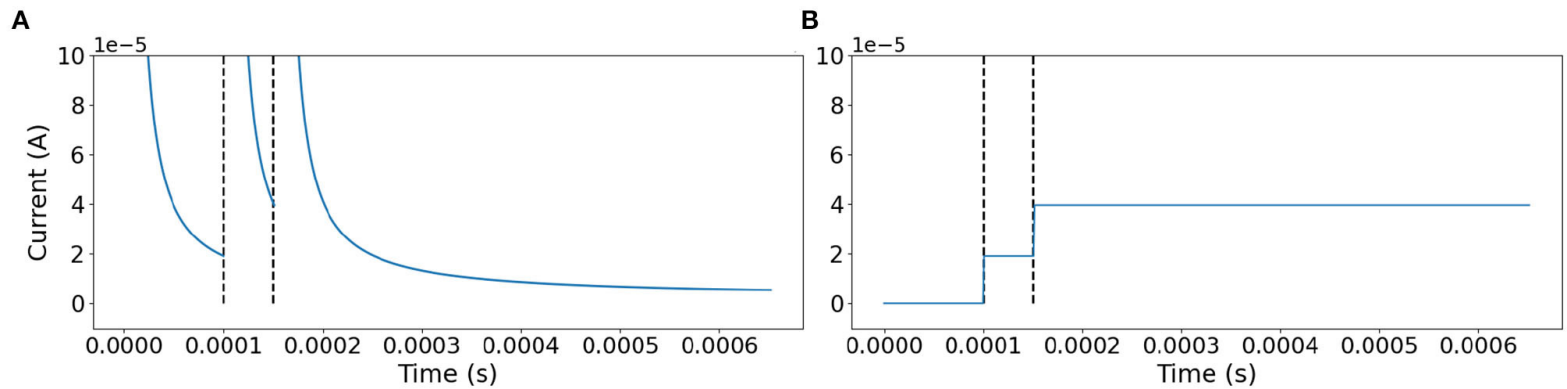
Signal rebuild scheme. **(A)** Behavior of Equation (7) evaluated at each step of the simulation. **(B)** At each spike Equation (7) is evaluated with *p* equal to the time elapsed from the last spike, and the value is preserved until the next event is reached. The dotted line marks the time of two spikes.

Where:


$$\begin{array}{l} fr=\frac{1}{\alpha p} \end{array}$$

with: *p* = *t*^*n*^−*t*^*n*−1^. *t*^*n*^ is the time of the *n*-*th* spike. In order to maintain values on a more convenient time-scale α = 1 × 10^4^ and β = 1 × 10^−6^ are added as scale factors. Equation (7) is evaluated at each spike and the value is held until the next spike occurs, refer to Figure 5B.

Observe that Equation (7) is a decaying exponential function; therefore, it is possible to define a circuit that reproduces this behavior by using the capacitor discharging dynamic in a commuted capacitor scheme working as follows. At each spike event, a low impedance branch quickly charges a capacitor during the refractory period of the SN unit. Once the refractory period concludes, the charging branch for the capacitor is open, and discharge becomes through a branch with fixed impedance such that the current on the capacitor has a behavior similar to Equation (7), refer to Figure 5A. Once a new spike event occurs, the current value of the capacitor is registered and held until the next spike event.

This scheme based on frequency shows a better performance than other strategies previously introduced (Dupeyroux et al., 2021; Guo et al., 2021) since it demonstrates good noise mitigation capacity employing only one neuron.

## 4. Experiments and Results

**Figure 6:**
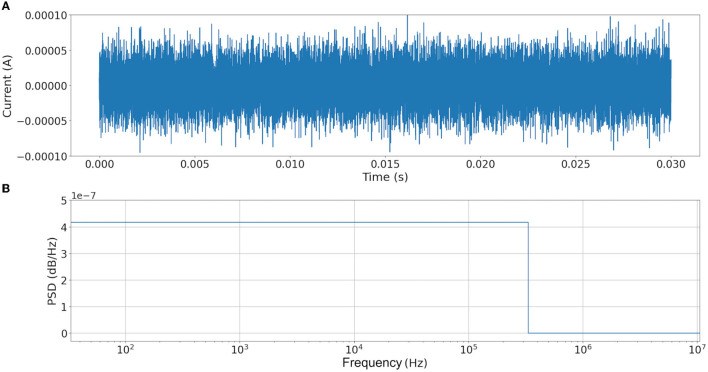
Synthetic white noise generate for this study. **(A)** Noise signal added. **(B)** Fast Fourier Transform of noise. It is possible to observe that the noise has a frequency uniform distribution between 1Hz and 31.6228 × 10^4^ Hz.

**Figure 7:**
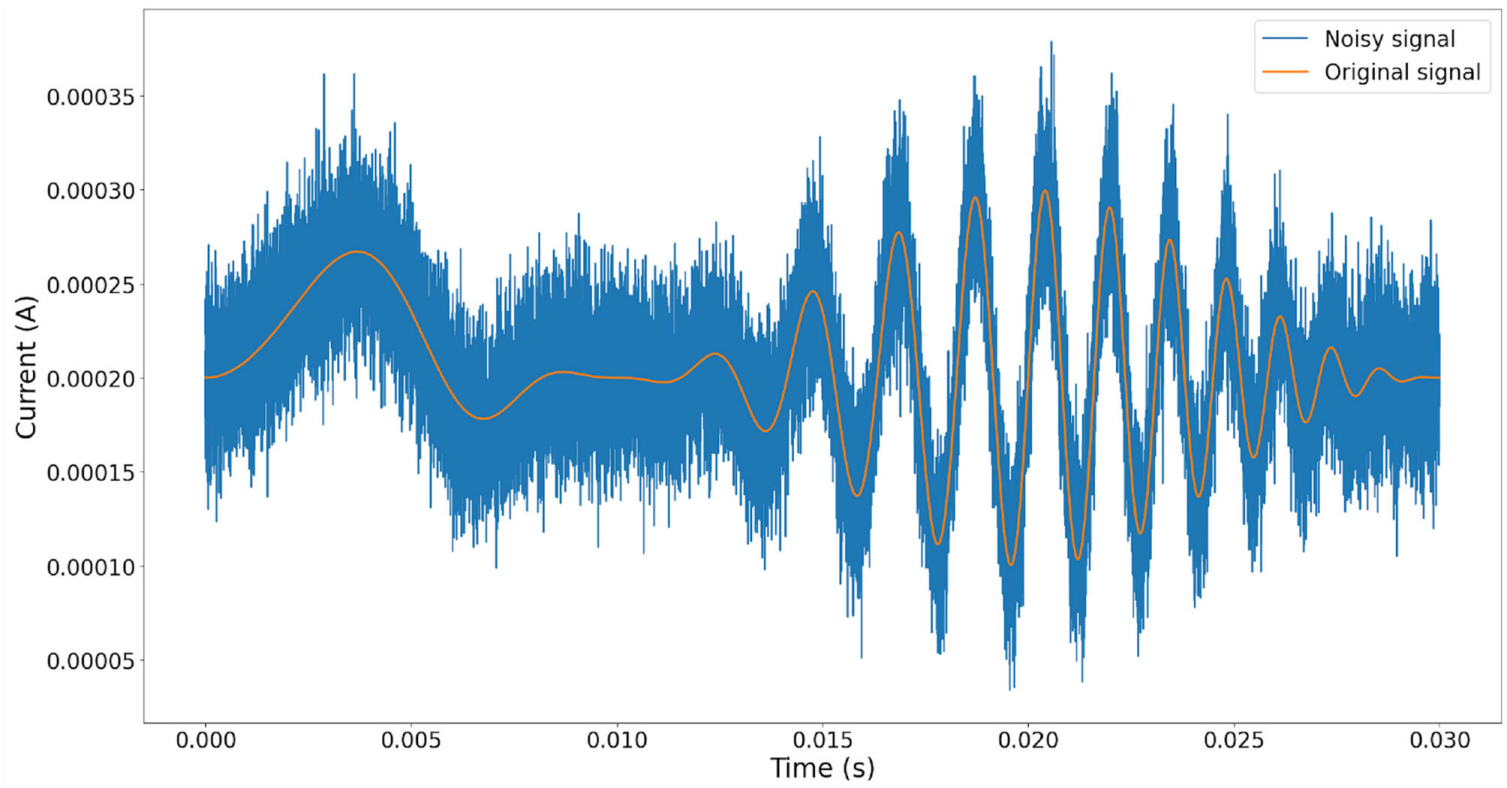
Comparison between the original signal with the noisy signal. For this experiment, the signal was computed evaluating the function $\frac{sin\left(1500t^{2}\right)cos\left(50t\right)}{50\;\times \;10^{6}}$ +200 ×10^−6^. It is important to observe that the signal must be positive at any moment.

**Figure 8:**
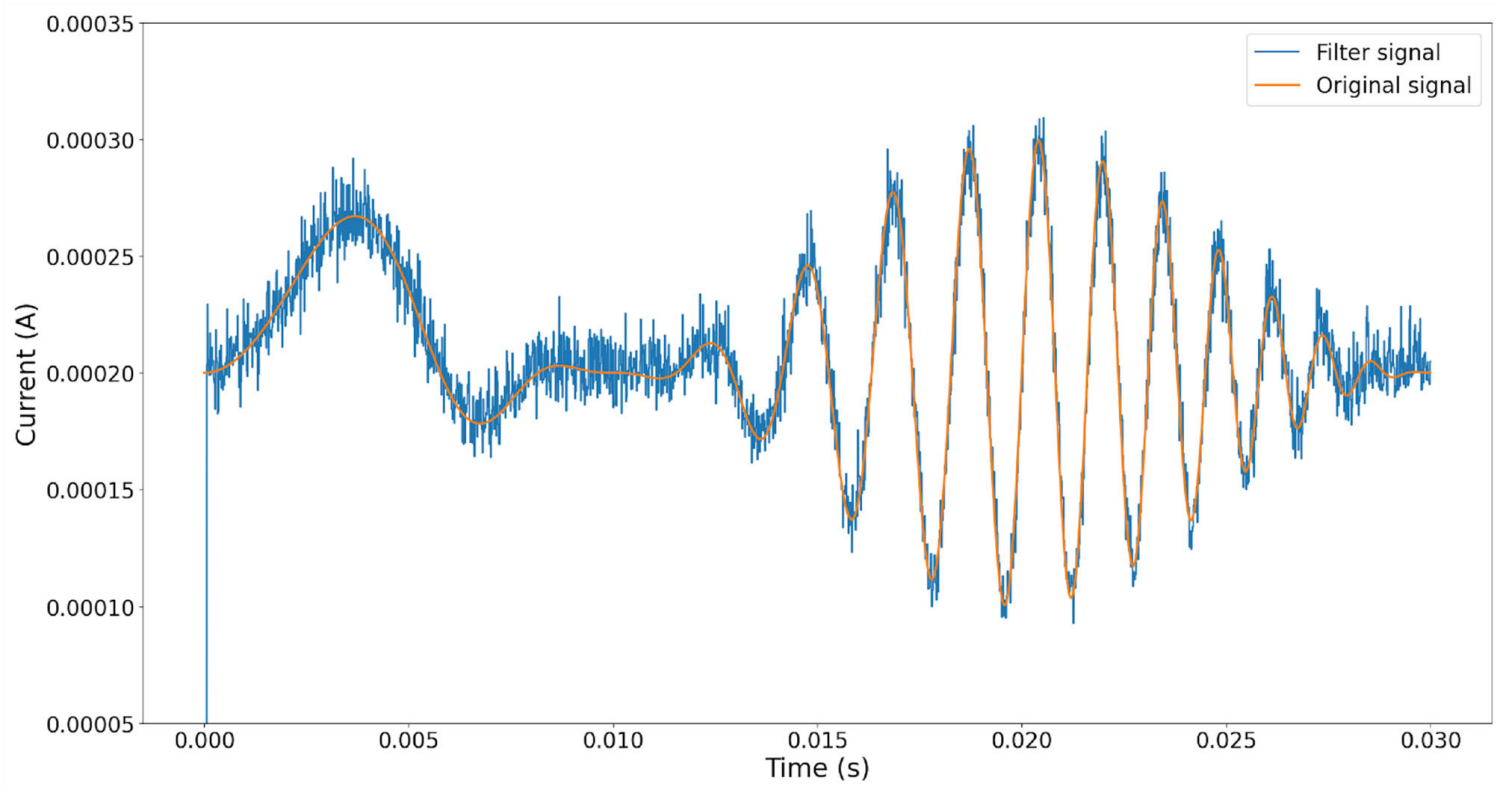
Comparison between signal rebuild from spike frequency on neuron output (blue line) and original signal (orange line).

To demonstrate the performance of our proposal, the following experiment was conducted. First, synthetic white noise is simulated to ensure a critical noise condition affectation over the clean signal with a uniform frequency distribution (Grinsted, 2022) (Figure 6). Second, the white noise is added to an arbitrary signal, refer to Figure 7. Finally, the noisy signal is used as the input for Equation (1), and the equivalent current output is computed using Equation (7). The results are shown in Figure 8.

**Figure 9:**
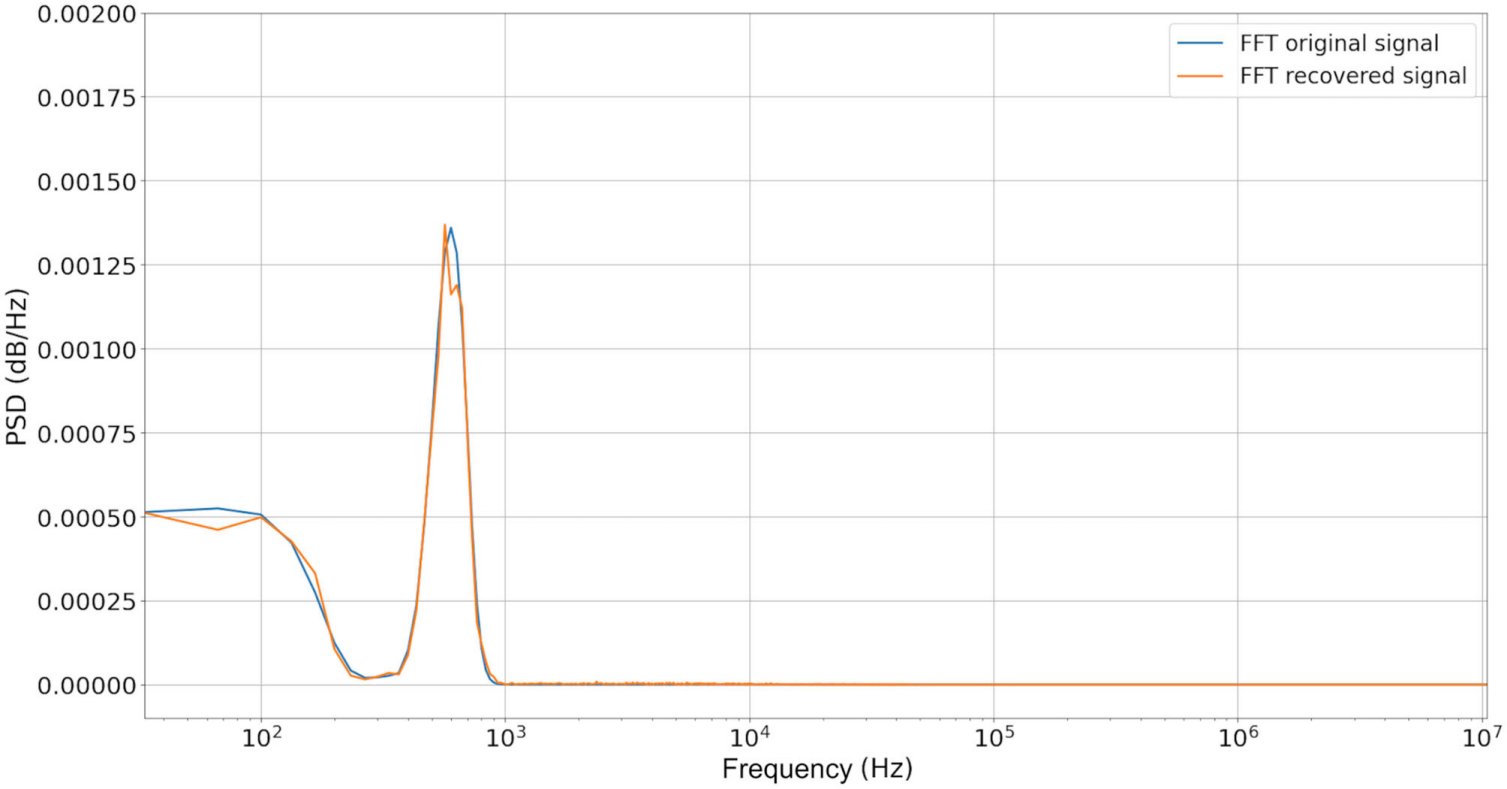
Power Spectral Density graph of the original signal and filter signal by our proposal. We can observe than primary frequencies are presented with low degradation.

It is possible to appreciate a significant noise reduction after the rebuilding operation. Figure 9 shows the Power Spectral Density of both original and noisy signals, and preservation of fundamental frequency is observed, thus we could conclude that the recovered signal is a good approximation of the original one. Notice that fundamental frequencies of the original signal are within the frequency range of noise.

**Figure 10:**
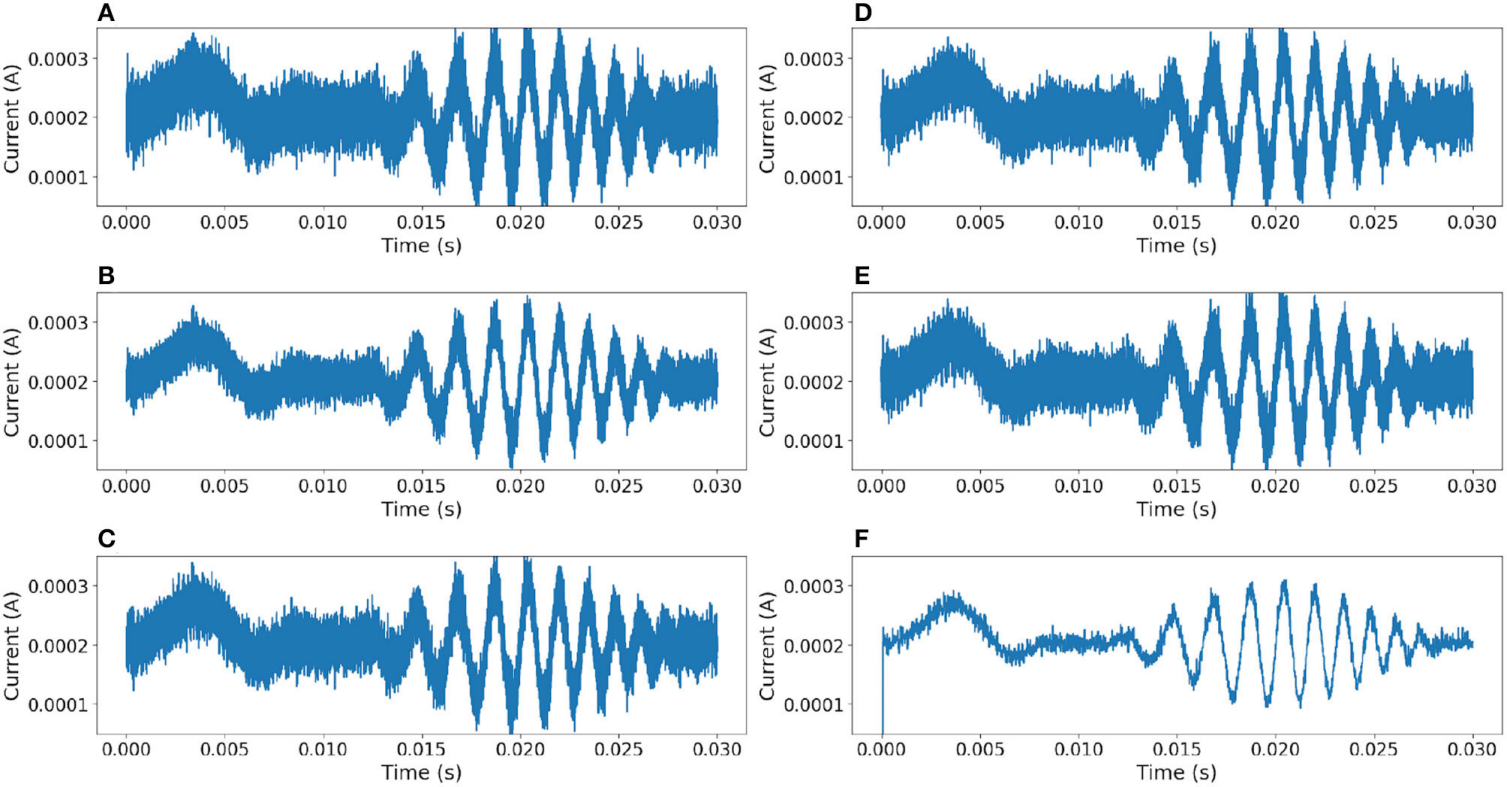
Comparison of results between different filters. **(A)** Median filter. **(B)** 3rd-order Chebyshev type 1 filter with cutoff frequency at 55*kHz*. **(C)** 3rd-order Chebyshev type 2 filter with cutoff frequency at 55*kHz*. **(D)** 3rd-order Butterworth filter with cutoff frequency at 55*kHz*. **(E)** 3rd-order Elliptic filter with cutoff frequency at 55*kHz*. **(F)** Neural filter proposed.

**Figure 11:**
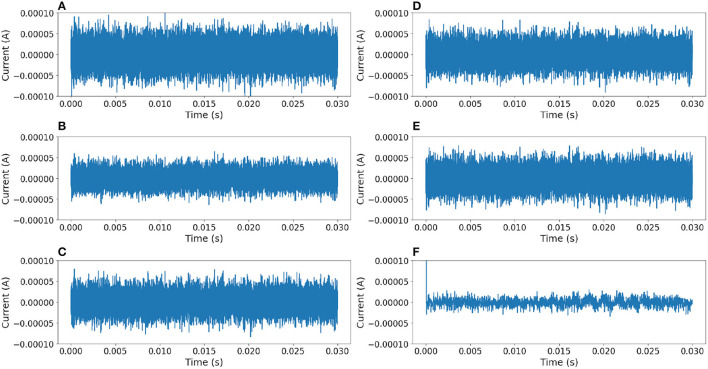
Comparison of error between outputs of different filters (additive white noise) and the original signal. The ideal error signal must be 0 at any time. **(A)** Median filter. **(B)** 3rd-order Chebyshev type 1 filter. **(C)** 3rd-order Chebyshev type 2 filter. **(D)** 3rd-order Butterworth filter. **(E)** 3rd-order Elliptic filter. **(F)** Neural filter proposed.

**Figure 12:**
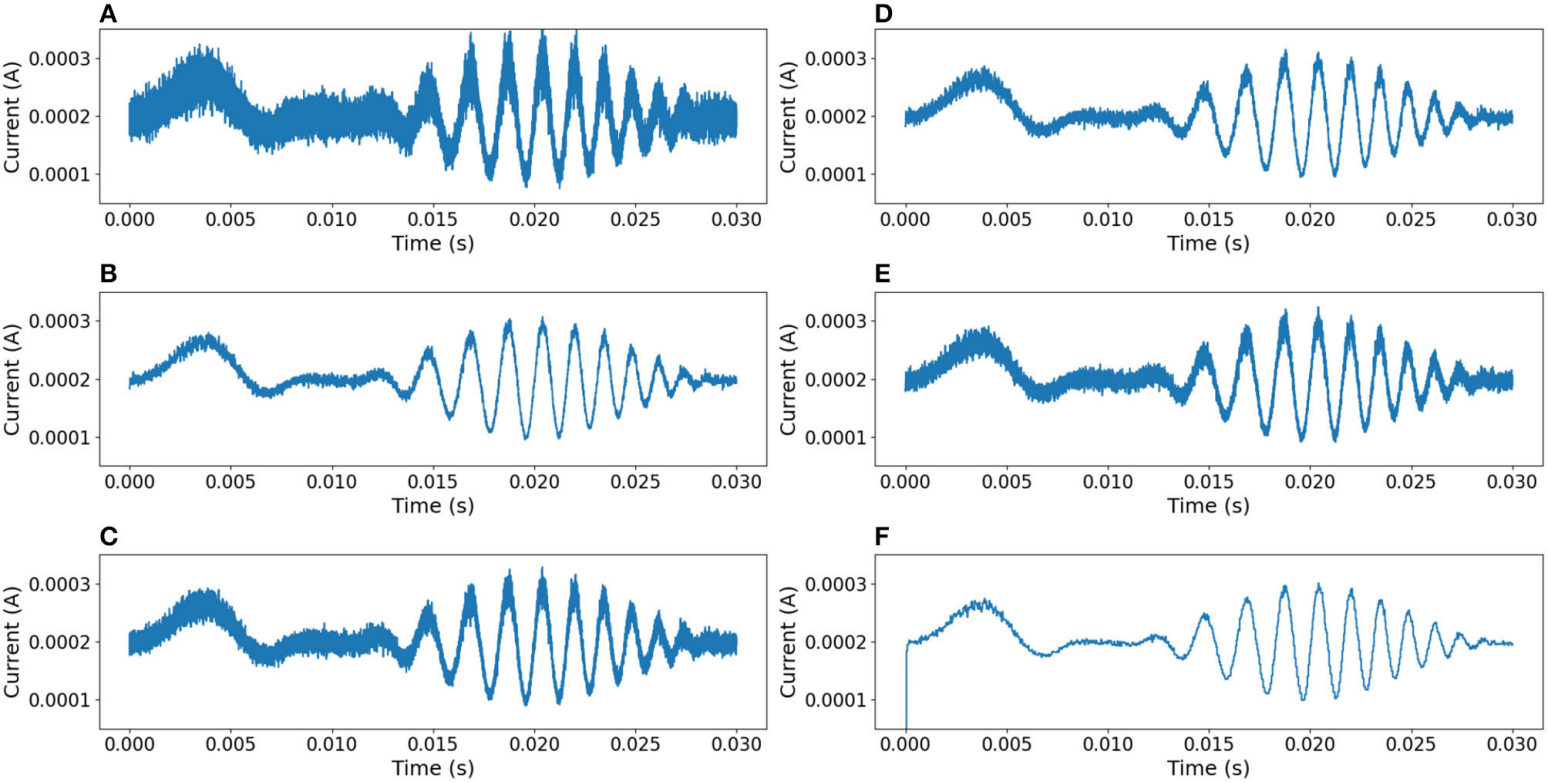
Comparison of results between different filters, using a signal affected by Gaussian Multiplicative Noise of 30% . **(A)** Median filter. **(B)** 3rd-order Chebyshev type 1 filter with cutoff frequency at 55*kHz*. **(C)** 3rd-order Chebyshev type 2 filter with cutoff frequency at 55*kHz*. **(D)** 3rd-order Butterworth filter with cutoff frequency at 55*kHz*. **(E)** 3rd-order Elliptic filter with cutoff frequency at 55*kHz*. **(F)** Neural filter proposed.

**Figure 13:**
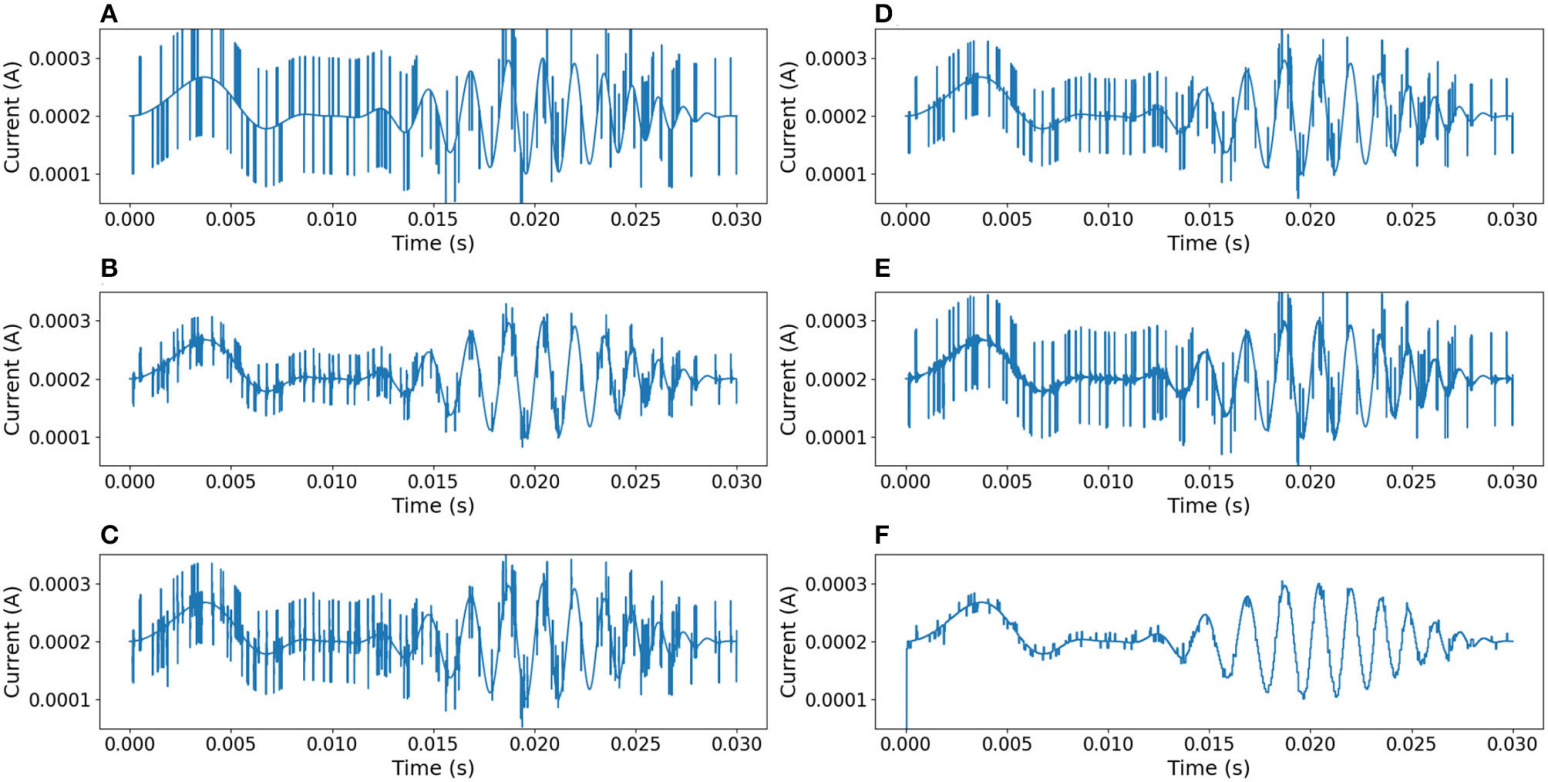
Comparison of results between different filters. The signal is affected by impulsive random noise (random pulses of 5μs and 0.1*mA* were added). **(A)** Median filter. **(B)** 3rd-order Chebyshev type 1 filter with cutoff frequency at 55*kHz*. **(C)** 3rd-order Chebyshev type 2 filter with cutoff frequency at 55*kHz*. **(D)** 3rd-order Butterworth filter with cutoff frequency at 55*kHz*. **(E)** 3rd-order Elliptic filter with cutoff frequency at 55*kHz*. **(F)** Neural filter proposed.

In order to compare this proposal with other approaches, the same noisy signal was filtered using linear (Chebyshev, Butterworth Butterworth, 1930, Elliptic) and digital (median Tukey, 1977) filters (Virtanen et al., 2020). Figure 10 shows the output responses obtained from these standard filters. Figure 11 shows the error measure of each filter, computed as the difference between the original and the output of the corresponding filter. Additional experiments were conducted using Gaussian Multiplicative Noise and Impulsive Random Noise, results are shown in Figures 12, 13.

To define a figure of merit for the proposed filter, we measure the distance between each filter response and the original signal (noiseless signal). As each filter introduces a different amount of time delay caused by the filtering process, Euclidean distance is not an appropriate choice since filtering techniques with a minimum delay will tend to render better results. Therefore, Fast Dynamic Time Wrapping (Salvador and Chan, 2007) (FastDTW) was used as a performance evaluation criterion. Comparisons of error results applying both Euclidean distance and FastDTW are shown in Table 1.

**Table 1:** Comparison between filter performance with Additive White Noise, Multiplicative Gaussian Noise, and Impulsive Random Noise.

**Filter**	**Euclidean**	**FDTW**	**MSE**	PSNR*
**Additive white noise**				
Median filter	0.257062	2.66536	4.40539 × 10^−09^	15.6014
3^*rd*^-order Chebyshevtype-1 filter	0.0300501	0.246951	6.02007 × 10^−11^	34.2452
3^*rd*^-order Chebyshevtype-2 filter	0.0575698	0.576117	2.20952 × 10^−10^	28.5982
3^*rd*^-order Butterworth filter	0.0378712	0.342871	9.56154 × 10^−11^	32.2359
Elliptic filter	0.047365	0.463987	1.49563 × 10^−10^	30.293
Proposed Neural filter	0.043605	0.187784	1.26760× 10^−10^	31.0114
**Multiplicative gaussian noise**				
Median filter	0.0574893	0.543259	2.20335× 10^−10^	28.6104
3^*rd*^-order Chebyshevtype-1 filter	0.0278647	0.153496	5.17626× 10^−11^	34.901
3^*rd*^-order Chebyshevtype-2 filter	0.0365451	0.299232	8.90364× 10^−11^	32.5455
3^*rd*^-order Butterworth filter	0.0306838	0.206129	6.27664× 10^−11^	34.0639
Elliptic filter	0.0338538	0.260648	7.64055× 10^−11^	33.21
Proposed neural filter	0.0453503	0.170839	1.37110× 10^−10^	30.6705
**Impulsive Random Noise**				
Median filter	0.0653834	0.142627	2.85000× 10^−10^	27.4928
3^*rd*^-order Chebyshevtype-1 filter	0.0364574	0.172277	8.86096× 10^−11^	32.5664
3^*rd*^-order Chebyshevtype-2 filter	0.0488858	0.20908	1.59321× 10^−10^	30.0185
3^*rd*^-order Butterworth filter	0.0468088	0.160894	1.46071× 10^−10^	30.3956
Elliptic filter	0.0486396	0.226337	1.57720× 10^−10^	30.0623
Proposed Neural filter	0.0438345	0.173065	1.28098× 10^−10^	30.9658

Highlighted values represent the best performance according to the used criterion. A lower value implies better performance (*Higher value is better). The results shown in this table are the average values obtained from 15 conducted simulations.

## 5. Conclusion

This study introduced the capacity and performance of simulated spiking neural network circuits to recognize primary signal information from signals corrupted deliberately with noise. Our proposal works as the analog mobile mean filter (refer to Mean Value Theorem for Integrals section) minimizing digital electronics, thus reducing the required number of transistors. Our frequency base decoding scheme has proven to have a good noise rejection, specially added white noise, but maintaining good performance with other types of noise, bringing artificial intelligence closer to circuit technology to deliver innovative solutions to filter white noise with the same frequency domain as the original signal, with minimal latency and low information loss. It is also a promising approach to, i.e., the conception of future innovative lab-on-chip implementations. Increasing the signal-to-noise ratio rejection ratio, cost efficiency, and sensitivity, is essential in these devices.

## Data Availability Statement

The raw data supporting the conclusions of this article will be made available by the authors, without undue reservation.

## Author Contributions

LG-S proposed, developed, programmed the neural filter code, conducted the simulation runs, and wrote the first draft of the manuscript. VP-P and HS proposed modifications to the encoding and decoding strategy architectures. ER-E reviewed the test bench for the filter experiments. JM-N helped with the neural modeling in Python. All authors contributed to the conception and design of the study, manuscript revision, read, and approved the submitted version.

## Funding

The authors would like to thank the economic support of the projects SIP 20210124, 20221780, 20211657, 20220268, 20212044, 20221089, 20210788, 20220226, and COFAA and CONACYT FORDECYT-PRONACES 6005.

## Conflict of Interest

The authors declare that the research was conducted in the absence of any commercial or financial relationships that could be construed as a potential conflict of interest. The reviewer AZ declared a shared affiliation with the authors to the handling editor at the time of review.

## Publisher's Note

All claims expressed in this article are solely those of the authors and do not necessarily represent those of their affiliated organizations, or those of the publisher, the editors and the reviewers. Any product that may be evaluated in this article, or claim that may be made by its manufacturer, is not guaranteed or endorsed by the publisher.
